# The Determinants of Reported Personal and Household Hygiene Behaviour: A Multi-Country Study

**DOI:** 10.1371/journal.pone.0159551

**Published:** 2016-08-19

**Authors:** Robert Aunger, Katie Greenland, George Ploubidis, Wolf Schmidt, John Oxford, Valerie Curtis

**Affiliations:** 1 Environmental Health Group, London School of Hygiene and Tropical Medicine, London, England; 2 Institute of Education, University College London, London, England; 3 School of Medicine, Queen Mary, University of London, London, England; University of Otago, NEW ZEALAND

## Abstract

A substantial proportion of the total infectious disease burden world-wide is due to person-to-person spread of pathogens within households. A questionnaire-based survey on the determinants of hand-washing with soap and cleaning of household surfaces was conducted in at least 1000 households in each of twelve countries across the world (N = 12,239). A structural equation model of hygiene behaviour and its consequences derived from theory was then estimated on this dataset for both behaviours, using a maximum likelihood procedure. The analysis showed that the frequency of handwashing with soap is significantly related to how automatically it is performed, and whether or not someone is busy, or tired. Surface cleaning was strongly linked to possessing a cleaning routine, the perception that one is living in a dirty environment and that others are doing the behaviour, whether one has a strong sense of contamination, as well as a felt need to keep one’s surroundings tidy. Being concerned with good manners is also linked to the performance of both behaviours. This study is the first to identify the role of manners, orderliness and routine on hygiene behaviours globally. Such findings should prove helpful in designing programs to improve domestic hygiene practices.

## Introduction

A substantial proportion of the total infectious disease burden world-wide is due to person-to-person spread of pathogens within households. Person-to-person transmission in the home can occur by direct hand-to-mouth transfer, via food prepared in the home by an infected person, or by transmission due to aerosolised particles resulting from sneezing, vomiting or fluid diarrhea [1–5]. Apart from transmission by inhalation of airborne particles, these infections are preventable by good hygiene practices. Hand and surface hygiene play a part in reducing the spread of not only colds but also influenza. [6, 7] Personal and household hygiene can also serve as a defensive strategy against future epidemics. [8] Hygiene is therefore important as a first line of defense to mitigate the spread of pathogens in people’s everyday environments. [2, 9] Hand-washing with soap is probably one of the most important means of preventing infectious disease transmission; systematic reviews show it can reduce rates of diarrhoeal disease by 30–47% [10–12] and rates of respiratory infection by 23% [13]—both of which are among the top five causes of death globally. (WHO 2008) Hand-washing with soap at key times can also prevent the transmission of hospital-acquired infections [14, 15] and influenza. [16]

Similarly, ensuring that household surfaces remain clean can be important. Preventing food-related infections, for example, relies on a combination of good hygiene practices during food preparation, cooking and storage. An important preventative measure in this context is the regular cleaning of household surfaces in kitchens and bathrooms (‘fomites’). [17, 18] For example, a study in the UK showed 12% of household surfaces were contaminated with the vaccine strain of polio virus in households where a child had just been immunized, giving an indication of how easily viruses can spread from child faeces. [19] In a developing country context, food can become infected with human pathogens [20]–and even children’s toys can be a significant contamination risk. [21, 22] Reducing the presence of pathogens on fomites can thus have an impact on health. [9, 23–25]

Perhaps the two most important behaviours for alleviating domestic infectious disease burden world-wide, then, are hand-washing with soap, and surface cleaning. However, even in the UK, hand-washing rates, even after toilet use, are low–below 70% for females and 50% for males. [26] A variety of studies have also shown that household surfaces ranging from computer keyboards, kitchen cutting boards, cleaning cloths and mobile phones can be considered reservoirs of infectious microbes, [27] suggesting a lack of safe household cleaning practices contribute to everyday disease transmission as well. [28] For this reason, the current study focuses on hand-washing with soap, and household surface cleaning.

### The Behaviour Determination Model

The objective of this study is to identify those factors which are most important in determining handwashing with soap and surface cleaning behaviour, to help inform future promotion efforts targeting these behaviours. To do this we developed a theoretical model, consistent with relevant theoretical and empirical literatures, of how these hygiene behaviors are determined. As the study was to take place at global scale, our approach was inclusive, to cover the largest possible range of factors that might be important.

**Fig 1:**
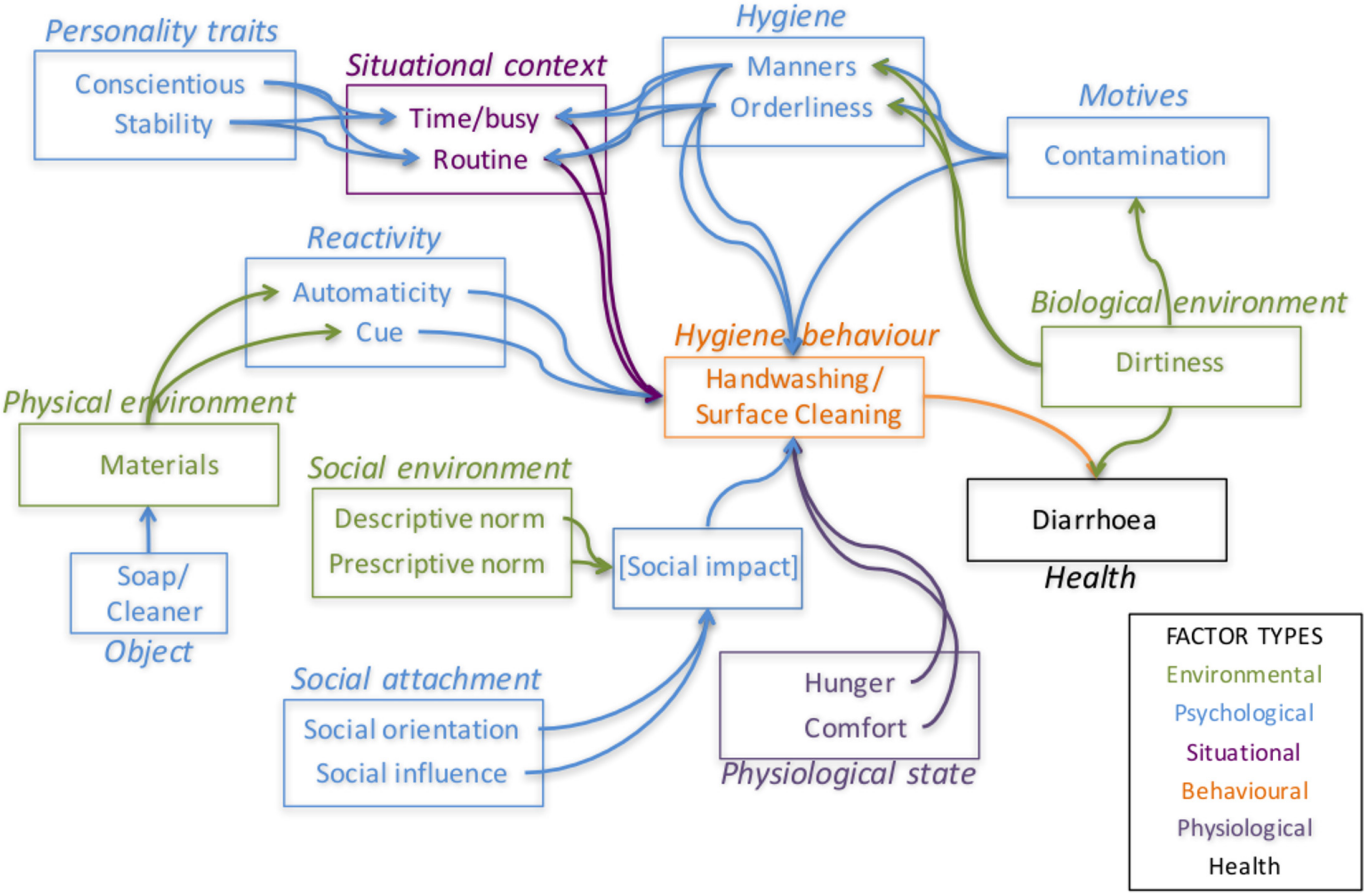
The Behaviour Determination Model.

Our Behaviour Determination Model is shown in Fig 1. It is based on variables specified in the Evo-Eco approach to behaviour determination developed by the authors. [29] The two behaviours of interest, handwashing with soap and surface cleaning, are shown in orange at the centre. We postulate several types of causes impact on these, based on theory in health psychology. Models in this field generally assume that the environment (in green in the figure) is an exogenous factor (i.e., exists independent of human action). [30] Environmental factors, in turn, influence psychological characteristics (in blue), which influence behavioural outcomes (in orange), which (finally) influence health status–particularly, in this case, infectious disease health (in black). This causal chain follows the convention from the most popular models of behaviour in cognitive and health psychology, such as the Theory of Planned Behaviour, [31], the Fishbein Integrated Model [32], and Social Cognitive Theory [33]. One conceptual complication is that some factors in the model are considered to be situational (in purple), as ‘in the moment’ amalgams of psychological pressures and physical constraints on behaviour (e.g., being temporally rushed due to an ambitious ‘to do’ agenda for the day). Situational constraints have been emphasized by ecological models of behaviour [34, 35] as well as by the person-situation debate in social psychology. [36–38]

Variables taking part in this causal sequence have also been broken down–for reasons of analytic tractability–into four causal ‘streams’, each of which replicates the environment-psychology-behaviour-health chain, but in different domains: physical, social, biological and situational. [39] Each of these streams is assumed to have independent effects on the target behaviours. Practice of the target behaviours is then assumed to have an impact on health, in terms of frequency of diarrhea.

The first stream begins in the Physical Environment, and notes that hygiene behaviours require specific physical infrastructure to take place–soap and water for handwashing with soap, and surface cleaner for cleaning surfaces. It is assumed that perception of these materials in the environment can serve as cues to the performance of hygiene behaviours, based on previous studies of handwashing. [40]

The second stream begins with the social environment, where social norms are a key influence on behaviour. These norms come in two forms: Descriptive and Prescriptive. Descriptive Norms concern the perception of how prevalent a practice is in one’s familiar social world; Prescriptive Norms concern whether the informant thinks others care about whether a practice is performed. However, the effect of these social norms on behaviour is moderated by an individual’s degree of attachment to the social world, as measured by their Social Orientation and Social Influence. [41–43] In effect, perceptions about how important social others themselves perceive disease threats are seen as influencing one’s own norms about whether to engage in hygiene behaviour. The net effect of this interaction is called ‘Social impact’ here (inferred statistically as a latent variable), which is the immediate effect of all social factors on hygiene behaviour.

The third, Biological, stream begins in a ‘dirty’ environment (consisting of disease threats) that might inspire hygienic behaviour through a sense of contamination, as well as specific hygiene motivations (manners and orderliness), which can impact on hygiene behaviour. [39] While manners and orderliness have not been well-studied in the academic psychological or health promotion literatures, we believe they warrant inclusion based on hypothesized links in the anthropological and sociological literatures between contamination and a desire not to infect others with one’s own pathogens (which we call ‘manners’) [44–46] and a more general sense of tidiness or order in the environment. [47]

Finally, two causal streams consist of situational factors. One is strictly based on psychological factors associated with physiological states that might impact directly on behaviour: a state of hunger or discomfort (for example, dirt on hands). As these are situational states, they are coloured purple in the figure. Other situational effects are due to temporal constraints such as being busy, or having established a regular routine at some point in the day for performing the target behaviours. [48–50]

The situational effects are assumed to be moderated by different types of personality–again, a suggestion from the person-situation debate literature. [38, 51, 52] The dominant model of personality is the ‘Big 5’ model [53], with five dimensions: Conscientiousness (high levels of thoughtfulness, with good impulse control, organized and mindful of details), stability or neuroticism (emotional instability, anxiety, moodiness, irritability, and sadness), Openness (imagination and insight, with a broad range of interests), Extraversion (excitability, sociability, talkativeness, assertiveness, and high amounts of emotional expressiveness) and Agreeableness (trust, altruism, kindness, affection, and other prosocial behaviors). There appear to be personality types that are fearful of danger and disease, which might correspond to the personality type known as neurotic. [54, 55] We also hypothesize that individuals with a conscientious personality might take more care over applying rules of personal and domestic hygiene. Conscientious and Stable people react differently to a sense of being busy or tired, compared to individuals with other personality types, persisting in performing hygiene behaviours. Having a strong sense of manner or orderliness can also ameliorate a tendency to rest or ‘forget’ to wash hands when tired or busy. (Orderliness, though not one of the ‘Big Five’ personality traits, can also be thought of as a personal trait–in particular, a preference for a neat and orderly domestic (and work) environment.)

Finally, we have also included the Object needed to engage in the relevant behaviour, like soap or surface cleaner, as an explicit factor facilitating behaviour. [56] Positive attitudes toward this Object are assumed to influence the probability that a household will have that object and other infrastructural support in place for the behaviour to occur.

## Methods

### Ethics statement

Data collection was carried out in January and February, 2011, by Opinion Matters, an international consumer research agency based in London. All participants contacted directly (i.e., face-to-face) provided written consent; those participating on-line indicated their consent by clicking on a specific button on the initial web-page associated with the study, while those contacted via telephone provided verbal consent, which was noted in interview records. All data collection protocols and research design were approved by the Ethics Committee of the London School of Hygiene and Tropical Medicine. All participants consented to scientific use of their responses prior to completing the survey questionnaire. The original data will be made available to anyone who requests access. (Requests can be made directly to the second author).

### Questionnaire design

Questions were designed to address each of the factors in the model from Fig 1. The questionnaire was composed of 119 questions, plus 11 background questions about the age, gender, educational attainment and occupation of the respondent, as well as the regional location (within country), demographic and material composition of their household. (See Appendix 1.)

The questionnaire was written in English and translated into the dominant mother-tongue and additional languages, where necessary, in non English-speaking countries by professional natives based in the country of the target language (i.e., French in France; German in Germany; Portuguese in Brazil; Hindi and English in India; Arabic and English in Saudi Arabia and UAE; Bahasa and English in Malaysia; French and English in Canada; Simplified and Traditional Chinese in China). All translations were carried out by one native speaker of the relevant country and then proof-read by a second professional native translator. Translators were also asked to make amendments to the questionnaire (if necessary) to incorporate local knowledge and customs (e.g., demographic nuances) and provided English translations for any of these changes. Respondents were asked at the beginning of the questionnaire which language they would like to be interviewed in.

### Sampling

Twelve countries were chosen to represent each of the seven continents (UK, USA, Canada, France, Germany, Australia, South Africa, Malaysia, Brazil, Middle East) with the addition of the two most populated countries in the world (China and India). A sample size of 1000 per country was judged sufficient to establish representative patterns of response, as analyses would not involve more than twenty-five variables simultaneously, and 30 samples per variable is typically more than sufficient to achieve the standard level of statistical power to test hypotheses (i.e., tests with p < 0.05). Within-country samples were collected to reflect splits of gender, age, household income and geographical region, based on WHO data on each country’s population profile for these variables, so that each country’s sample was representative of the overall population in that country. All households were also required to have ready access to a water source as a simple precondition to perform the target behaviours.

### Collection methods

For cost-efficiency purposes, identification of respondents through on-line methods was preferred. However, in some countries, not all respondents could not be identified in this way, as lower income respondents often do not have access to the web. To make up a representative sample in these cases, potential participants for telephone or face-to-face interviews were identified at point of contact according to income. The questionnaire was thus delivered through one of three means: online via an email invitation, via computer-assisted telephone interview (CATI), or face-to-face (see Table 1). In each case, question order was randomized in the actual delivery of the questionnaire (with the exception of blocks of questions with a particular format, such as the personality test, and the background data section), in order to ensure that any biasing effects of prior priming of one question on another could be minimized. We note that reviews find little influence of the means of administering questionnaires on reporting biases, which tend to be consistent across such methods. [57, 58]

**Table 1:** Breakdown of data collection methods per country.

	Online	Telephone	Face-to-face
**Australia**	1010		
**Brazil**	859	203	
**Canada**	1014		
**China**	880	200	
**France**	1027		
**Germany**	1005		
**India**	907	102	
**Malaysia**	754	250	
**Middle East**			1003
**South Africa**	808		200
**UK**	1010		
**USA**	1009		

#### Online

Potential participants for online responses were identified using both the Opinion Matters online panel as well as trusted partners that adhere to the same strict codes of conduct and research guidelines. These panels are actively-managed online global panels recruited for market research purposes. All panelists have gone through a double opt-in process and have agreed to participate in paid online surveys, and to provide honest opinions for market research studies. A wide range of recruitment processes are used to generate the panel including referral, Web advertising and public relations, to partner-recruited panels and alliances with heavily trafficked web portals.

Potential panelists were sent an invitation to participate in the survey via email, on a random basis within the target groups for the research. A large number of respondents were eliminated by algorithms in the Globalpark software as the data collection process proceeded, using the following criteria:

- those whose answers were abnormally patterned (e.g., used the same response for a majority of questions, or answered the questionnaire too quickly, based on the average speed of questionnaire completion)
- those missing a single response, either demographic or in the main questionnaire
- those screened out by not fitting into demographic quotas (i.e., respondents have to fulfil the demographic criteria to determine eligibility, and even if eligible, whether the quota for their profile is already full, before they can complete the questionnaire proper).

Respondents who fully completed questionnaires received points worth about US$3.00 which could then be redeemed for money or against charitable donations. Safeguards ensured that respondents could not try to complete the questionnaire if it was not appropriate (i.e., they could not proceed unless they had ticked an answer for all questions on each page; if there were alerts for contradictory answers, and so on). Online responses took an average of 20 minutes to complete.

#### Telephone

In some countries, it wasn’t possible to rely on panels or on-line respondents to achieve a sample representative of the country’s population. In the cases of Brazil, China, India and Malaysia, datasets were augmented by the inclusion of a component collected via a computer-assisted telephone interviewing (CATI) method. This involved random digit dialling. Unlike the online case, no incentive was used for these respondents. All such interviews were conducted by native language interviewers in each country using CATI-SPSS Dimensions software.

#### Face-to-Face

In the case of the Middle East and South Africa, face-to-face interviews were conducted because some segments of the population were known not to own land-line telephones, and hence could not be effectively reached via the CATI method. The way the personal interviews were conducted varied slightly between these two territories. In South Africa, interviewers targeted low-income areas to fulfill the quota stipulations and respondents were then randomly selected in that area. People were approached in the street or a public place, asked the screening questions to verify that they met the screening criteria. Respondents were interviewed and their responses were recorded on paper questionnaires and later inputted into the online research platform. Respondents were incentivised with R30 (approx. 4 USD). Interviews collected in this way lasted an average of 15 to 20 minutes.

In the Middle East, interviewers were briefed on the project and performed mock interviews before the beginning of the project. Every interviewer was given a laptop with USB internet connection enabling him/her to conduct the interview from any location. Interviews were therefore carried out at respondents’ homes, coffee shops, malls, internet cafés, or at universities (especially for younger age groups). In principle, incentives were not offered for such interviews; however, in some cases (where interviews were done outside the home), interviewers offered the respondent a cup of coffee, juice or cake. The average interview length varied between 25 to 35 minutes.

#### Data transcription

Online responses were exported from an integrated research platform (Globalpark) by Opinion Matters personnel into a survey reporting software program (SNAP), and from thence were converted into an Excel spreadsheet. Interviewers recorded telephone and face-to-face responses by hand onto printed data-sheets which were then transcribed into electronic records in an Excel database using codes consistent with the on-line records.

## Data Analysis

### Analytic strategy

#### Variable construction

Variables entering into the analysis were calculated in various ways, depending on the nature of the phenomenon being measured (see Table 2). Some variables were binary (e.g., gender). In most cases, a scale value was created based on the Likert scale responses (e.g., strongly agree to strongly disagree). In a few cases, indices were created by combining responses from multiple Likert scale questions on the same topic, if aggregate cross-correlation values achieved a standard level of significance (i.e., Cronbach’s alpha > 0.6). Because the surveys were restricted to reported behaviour, which is notoriously subject to courtesy and other biases, [59–61] the likelihood of engaging in each of the target hygiene behaviours was constructed from a variety of item responses using Polychoric Principal Component Analysis, a data reduction method that can be used for variables that are ordered categorically.

**Table 2:** Study Variables.

Variable Description	Variable Definition	Questionnaire Items	Calculation Method/Coding*	Variable Group	Direction of Effect
**Dirty environment**	Perception of environmental contamination	• The neighbourhood where I live is quite dirty. (Q2) • If you walk around the neighbourhood where I live, you will see human or animal faeces on the ground. (Q3)	Index (Cronbach’s alpha = 0.70)	Environment (Biological)	High score = dirtier environment
**Material availability**	Perception that all material requirements for performing the target behaviour are available	• **HW**: All the things I need to wash my hands with soap are readily available in my house (i.e. clean water and soap). (Q24) • **SC**: All the things I need to clean surfaces are readily available in my house (i.e. cleaning liquid and a cloth). (Q25)	––	Environment (Physical)	High score = material available
**Social Orientation**	Degree of reliance on others to get things done	• I don’t have a strong group of friends who help me if I have a problem. (Q4) • I don’t often interact with other people. (Q5)	Index	Social Attachment	High score = social person
**Social influence**	Degree of responsiveness to perceived social pressure	• I do what I like; I really do not care what other people say about me. (Q6) • In general, I want to do what the people who are important to me think I should do. (Q7)	Index (Q6 reverse-scored)	Social Attachment	High score = more dependent person
**Time/busy factor**	Perception of the degree to which performance of target behaviours intrudes into daily life	• **HW**: Even when I am tired, I manage to wash my hands with soap after the toilet. (Q19) Even if I am busy, I manage to wash my hands with soap after the toilet. (Q20) • It takes too much time to wash my hands with soap each time I prepare food. (Q21) • Hand-washing with soap is quick and easy to do. (Q23) • **SC**: It takes too much time to wash kitchen surfaces with a cleaning product each time I use them to prepare food. (Q22)	HW: PCA score	Situational Context	High score = still does behaviour
**Hygiene routine**	Degree to which performance of target behavior is built into daily routines	• **HW**: I can easily go through my daily routine without washing my hands with soap. (Q32) • It’s easy to forget to wash your hands before eating food. (Q33) • **SC**: I try to make sure I do a bit of cleaning around the house every day. (Q34) • I try to regularly clean the kitchen thoroughly. (Q35) • I try to regularly clean the bathroom thoroughly. (Q36)	PCA score; items based on several from the Variety Assessment Scale, [64] and designed to measure how much individuals prefer a steady, consistent stream of activity during their daily lives.	Situational Context	High score = more routine
**Cue**	Degree to which initiation of target behaviour performance is perceived to be in response to an environmental cue	• **HW**: Seeing soap after having been to toilet makes me wash my hands. (Q69) • A bad smell or visible dirt on my hands makes me want to wash them with soap. (Q70) • **SC**: Seeing a bit of dirt on a household surface makes me want to clean it immediately. (Q75)	HW: Index	Reactivity	High score = acts upon cues
**Automaticity**	Degree to which target behaviour performance is perceived to be automatic	• **HW**: I sometimes start washing my hands with soap without even realizing I'm doing it. (Q65) • I feel strange when I don’t wash my hands with soap after using the toilet. (Q66) • Washing my hands with soap before I eat a meal is something I do automatically. (Q67) • **SC**: I sometimes start cleaning my house without even realizing I'm doing it. (Q71) • I feel strange when I don’t clean my house regularly. (Q72) • Washing the kitchen surface or chopping board with a cleaning product before I prepare food or eat a meal is something I do automatically. (Q73)	Index; questions derived from the automaticity sub-scale of the Self-Report Habit Index (SRHI) of Verplanken. [65] as applied to hand-washing with soap or surface cleaning	Reactivity	High score = high automaticity
**Descriptive norms**	Perception of degree to which performance of target behaviour is common in local area	• **HW**: I would say that hand-washing with soap is not something we practice much round here. (Q12) • **SC**: I would say that cleaning household surfaces is something we practice a lot around here. (Q11) • **AB**: I would say that hand-washing with an antibacterial product is not something we practice much around here. (Q13)	––(Q12, Q13 reverse-scored)	Social Environment	High score = more normative
**Prescriptive norms**	Perception of degree to which important others are thought to care about focal individual’s performance of the target behaviour	• **HW**: Most of the people important to me don’t care if I wash my hands with soap. (Q14) • **SC**: Most of the people important to me think I should keep my household surfaces clean. (Q15)	––(Q14 reverse-scored)	Social Environment	High score = others care more about own hygiene
**Orderliness**	Perceived degree of sensitivity to disorder in local environment	• If I see things in a jumble or out of place, I feel compelled to put them in order. (Q91) • I have a habit of constantly organizing the things in my house or at my work-place. (Q92) • I am a very tidy person (Q93)	PCA score	Hygiene	High score = more tidy
**Manners**	Reported degree of importance of hygiene manners	• I would shake hands knowing that my hands are dirty. (Q94) • As a guest in someone else’s house, I always make sure I clean up the room after using their toilet. (Q95) • I would not feel embarrassed if I sneezed or coughed in front of someone without covering my mouth. (Q96)	PCA score	Hygiene	High score = good manners
**Contamination sensitivity**	Reported sensitivity to perception of contamination	• If a home does not look dirty then it doesn’t need cleaning. (Q84) • Hidden germs cause diarrhea. (Q85) • After using the toilet there may be unseen contamination on my hands. (Q86) • You only need to wash your hands when they look or feel dirty. (Q87) • Using an antibacterial product to wash your hands does not provide a benefit over soap in regard to cleanliness. (Q88) • Hand washing technique is important to ensure all contamination is removed from hands. (Q89)	PCA score	Motives	High score = more sensitive
**Comfort**	Reported sensitivity to sense of physical discomfort	I’m not bothered by feeling sweaty and sticky. (Q59)	––	Motives	High score = cares about comfort
**Conscientiousness**	Degree to reported ability to organize and control everyday life	Dependable, self-disciplined. (Q39) Disorganized, careless. (Q44)	coded to derive values for one of the ‘Big 5’ personality dimensions [66]; this is a standard coding, hence it was made even though the alpha is low (alpha 0.34)	Personality Traits	High score = more conscientious
**Emotional Stability (Neuroticism)**		• Anxious, easily upset. (Q40) • Calm, emotionally stable. (Q45)	See above (alpha 0.43)	Personality Traits	High score = more stable
**Hunger**	Reported degree to which hunger inhibits performance of handwashing	If I am hungry, I often don’t bother to wash my hands before eating. (Q29)	––	Physiological State	High score = still washes hands
**Hand-washing behaviour**	Reported level of performance of handwashing with soap	• I washed my hands with soap ___ times yesterday. (Q97) • I wash my hands with soap after using the toilet. (Q98) • I wash my hands with soap before preparing or eating food. (Q99)	PCA score	Hygiene Behaviour	High score = more behavior
**Surface cleaning behaviour**	Reported level of performance of surface cleaning	• I spent time removing dirt from different places in my house yesterday. (Q101) • I [regularly] wipe kitchen and bathroom surfaces clean around my house. (Q102) • I use a cleaning product when I tidy my house. (Q103) • I used a surface cleaner ____ times in the past week. (Q104)	PCA score	Hygiene Behaviour	High score = more behaviour
**Hygiene object**	Attitudes toward products required to perform target behaviours	**HW**: Soap can be: ■ Disgusting ■ Comforting ■ Sexy ■ Purifying ■ A store of economic value ■ An indicator of social status ■ A means of caring for others ■ Something everyone uses ■ An object to play with ■ Something that helps me protect others from my germs ■ A badge of belonging to a social group ■ Other (Q82) **SC:** Surface cleaning liquid can be: ■ Disgusting ■ Comforting ■ Sexy ■ Purifying ■ A store of economic value ■ An indicator of social status ■ A means of caring for others ■ Something everyone usesAn object to play with ■ Something that helps me protect others from my germs ■ A badge of belonging to a social group ■ Other (Q83)	Index counting number of responses; values 0–11 (disgust was re-coded to 0 = disgusting, 1 = not disgusting to make it positive)	Object	High score = more positive attitude towards soap/liquid
**Illness frequency**	Reported frequence of diarrheal illness	I often get diarrhea. (Q108)	––	Infectious Health	High score = more disease

* Index = summation of scores from constituent question responses (i.e., based on aggregation of Likert scale responses)

PCA score = score from Polychoric Principal Components Analysis of the constituent question responses

#### Structural equation modelling

We used structural equation modeling (SEM) to test the Behavioural Determination model for hygiene behaviour. SEM involves estimating a multivariate model based on assumptions about specific causal relationships or dependencies between pairs of explanatory variables. [62] This is a powerful technique for analyzing the multiple causes of behavior identified in the Behaviour Determination model, as it is able to show relationships between causes as well as their relative significance with respect to the outcome variable, behaviour. In the present case, a random effects SEM was used in order to account for the likely dependency among participants from the same country. The variables are assumed to perfectly measure the theoretical concepts of interest (i.e., it is a purely structural, not measurement, structured equation model). That is, the analysis assumes that the latent variables perfectly measure what they indicate, so there is no need to separately estimate parameters other than those specified in the theoretical model (i.e., it is not exploratory factor analytic in nature).

Variables entered in the model were derived using Principal Components Analysis with a tetrachoric correlation matrix to reflect the ordinal nature of the items that comprised each construct, because a one-step SEM approach where the measurement and structural parts of the model would have been estimated simultaneously is not feasible due to memory constraints. We note however, that despite the fact that a one-step approach is theoretically more appropriate, in practice it returns similar results with two step approaches.

The Behaviour Determination Model was estimated simultaneously for handwashing with soap and surface cleaning (including observations with missing data). The estimated latent trait scores for all variables were entered into the analysis using the path analytic model as shown in Fig 1. All direct and indirect associations were jointly estimated. All reported model parameters were standardized so that their relative sizes could be compared.

Estimation was carried out with a maximum likelihood estimation procedure, using Mplus 5.21 software (http://www.statmodel.com/). Model fit was assessed with the Comparative Fit Index (CFI), the Tucker Lewis Index (TLI) and the Root Mean Square Error of Approximation (RMSEA). [63]

The final model was estimated in two steps. In the first step, a relatively large number of measures were used in the same categories. For example, three Motives were originally considered, based on many previous hygiene studies: nurture, social status, and disgust/contamination. [48] We also considered three measures of social environment: Social Orientation (how sociable and reliant on social contact an individual is); Social Influence (how susceptible to the opinions of others an individual is); and Social Communication (how much people in one’s social network talked about hygiene issues). All five of the ‘Big Five’ Personality traits were also included originally, as was Perceived Vulnerability to Disease (a psychological trait which measures the subjective impression of one’s constitutional ability to withstand infection [64]). However, in a second round of model estimation, only those variables proving significant in the first step were included in this estimation step. It is the results from this second step which are presented below.

Variation in the causation of these everyday behaviours between different countries is of some interest, but is the topic of a separate paper (as its description requires significant space), and so is not treated in the modelling performed here.

## Results

The survey included 12,239 participants in 12 countries. 42,331 respondents were approached, of whom 25,893 were screened out (see Data Cleaning section above), 4,197 dropped out and 12,239 (28.9% of invitees) completed the questionnaire. This sample accurately represents the population of the constituent countries by age, gender and income, although it is probably biased toward the more educated, as this was not one of the sample selection criteria (see Table 3).

**Table 3:** Characteristics of survey respondents.

	Survey Respondents N = 12,240 (n, %)
**Gender**	
**Male**	6178 (50.5)
**Female**	6062 (49.5)
**Age group**	
**16–24**	1544 (12.6)
**25–34**	3348 (27.4)
**35–44**	3021 (24.7)
**45–54**	2735 (22.3)
**55–64**	1194 (9.8)
**65+**	398 (3.3)
**Income**	
**Q1 (lowest)**	3090 (25.3)
**Q2**	3097 (25.3)
**Q3**	3086 (25.2)
**Q4**	2967 (24.2)
**Education level**	
**No formal education**	129 (1.1)
**Primary school**	269 (2.2)
**Secondary school**	4335 (35.8)
**Vocational training**	1587 (13.1)
**University degree**	4223 (34.9)
**Graduate degree**	1575 (13.0)

Table 4 provides the SEM numerical results. Included in the estimation process were a number of covariates: gender, age, employment status, income, education and children (presence/absence in the household). The strength of the relationships between variables in the Behaviour Determination Model for particular samples have also been depicted pictorially in SEM diagrams below. The greater the width of the connecting arrow, the larger the effect, as measured by the b estimate in Table 4 (with the range of values associated with each width being specified in the box at the bottom corner of each figure). All of the results depicted in the SEM diagrams control for the effects of the demographic variables just mentioned. The measures of fit for this model are TFI = 0.136; CFI = 0.349; RMSEA = 0.126.

**Table 4:** Structural equation model results.

	Two-Tailed			
	Estimate	S.E.	Est./S.E.	P-Value
**Diarrhoea**	ON			
**Children**	0.006	0.01	0.608	0.543
**Education**	-0.001	0.009	-0.141	0.888
**Gender**	0.063	0.009	6.917	< 0.001
**Age**	0.123	0.01	12.402	< 0.001
**Employed**	-0.008	0.009	-0.934	0.351
**Income**	0.031	0.009	3.413	0.001
**HW Behaviour**	0.056	0.009	5.894	< 0.001
**SC Behaviour**	0.041	0.01	4.233	< 0.001
**Dirty Environment**	-0.243	0.009	-27.787	< 0.001
**HW Behaviour**	ON			
**Children**	-0.015	0.008	-1.821	0.069
**Education**	0.022	0.008	2.874	0.004
**Gender**	0.023	0.008	2.963	0.003
**Age**	0.018	0.009	2.17	0.03
**Employed**	0.029	0.008	3.829	< 0.001
**Income**	0.035	0.008	4.592	< 0.001
**HW Cue**	-0.018	0.009	-1.98	0.048
**Hunger**	0.048	0.009	5.094	< 0.001
**Time/Busy**	-0.264	0.013	-20.941	< 0.001
**Contamination Sensitivity**	0.009	0.011	0.857	0.392
**Comfort**	-0.027	0.008	-3.336	0.001
**Manners**	-0.016	0.009	-1.796	0.073
**HW Prescriptive Norm**	-0.028	0.009	-3.106	0.002
**HW Personal Norm**	-0.133	0.011	-11.686	< 0.001
**Orderliness**	0.023	0.01	2.344	0.019
**HW Automaticity**	0.378	0.011	34.068	< 0.001
**HW Routine**	0.133	0.011	12.336	< 0.001
**HW Descriptive Norm**	-0.008	0.009	-0.961	0.337
**SC Behaviour**	ON			
**Children**	-0.059	0.008	-6.979	< 0.001
**Education**	0.029	0.008	3.823	< 0.001
**Gender**	0.08	0.008	10.136	< 0.001
**Age**	0.084	0.009	9.758	< 0.001
**Employed**	-0.006	0.008	-0.797	0.425
**Income**	0.052	0.008	6.688	< 0.001
**SC Cue**	-0.04	0.012	-3.32	0.001
**Hunger**	0.018	0.01	1.825	0.068
**Contamination Sensitivity**	0.059	0.011	5.395	< 0.001
**Comfort**	-0.021	0.008	-2.543	0.011
**Manners**	-0.019	0.009	-2.132	0.033
**SC Prescriptive Norm**	0.019	0.008	2.442	0.015
**Orderliness**	0.041	0.01	4.08	< 0.001
**SC Automaticity**	-0.251	0.015	-17.217	< 0.001
**SC Routine**	0.401	0.009	46.466	< 0.001
**SC Descriptive Norm**	0.055	0.009	6.101	< 0.001
**Anti-bac Soap**	ON			
**Children**	-0.04	0.01	-4.118	< 0.001
**Education**	0.04	0.009	4.555	< 0.001
**Gender**	-0.02	0.009	-2.212	0.027
**Age**	-0.106	0.01	-10.554	< 0.001
**Employed**	0.022	0.009	2.494	0.013
**Income**	0.008	0.009	0.883	0.377
**HW Cue**	-0.026	0.011	-2.475	0.013
**Hunger**	0.026	0.011	2.378	0.017
**Time/Busy**	0.096	0.016	6.183	< 0.001
**Contamination Sensitivity**	0.164	0.012	13.35	< 0.001
**Comfort**	-0.071	0.009	-7.465	< 0.001
**Manners**	-0.013	0.01	-1.219	0.223
**HW Prescriptive Norm**	-0.009	0.01	-0.834	0.404
**HW Personal Norm**	-0.058	0.013	-4.443	< 0.001
**Conscientiousness**	-0.056	0.011	-5.332	< 0.001
**Stability**	0.033	0.009	3.518	< 0.001
**Orderliness**	0.069	0.012	5.959	< 0.001
**HW Automaticity**	0.104	0.013	7.699	< 0.001
**HW Routine**	0.003	0.013	0.272	0.786
**Anti-bac Descriptive Norm**	-0.148	0.012	-12.484	< 0.001
**Manners**	ON			
**Dirty Environment**	-0.11	0.008	-13.253	< 0.001
**Time/Busy**	-0.036	0.01	-3.542	< 0.001
**HW Routine**	0.224	0.01	22.886	< 0.001
**SC Routine**	0.009	0.008	1.074	0.283
**Contamination Sensitivity**	0.353	0.01	36.101	< 0.001
**Orderliness**	ON			
**Dirty Environment**	0.092	0.009	10.193	< 0.001
**Time/Busy**	0.004	0.011	0.409	0.683
**HW Routine**	-0.009	0.01	-0.829	0.407
**SC Routine**	0.324	0.009	37.391	< 0.001
**Contamination Sensitivity**	0.245	0.011	22.997	< 0.001
**HW Materials**	ON			
**Soap**	0.106	0.015	7.32	< 0.001
**Cleaner**	0.08	0.015	5.517	< 0.001
**SC Materials**	ON			
**Soap**	0.082	0.015	5.663	< 0.001
**Cleaner**	0.106	0.015	7.323	< 0.001
**Contamination Sensitivity**	ON			
**Dirty Environment**	-0.197	0.009	-21.781	< 0.001
**HW Prescriptive Norm**	ON			
**Social Orientation**	-0.256	0.009	-29.044	< 0.001
**Social Influence**	-0.016	0.009	-1.81	0.07
**SC Prescriptive Norm**	ON			
**Social Orientation**	0.04	0.009	4.209	< 0.001
**Social Influence**	0.011	0.009	1.219	0.223
**HW Personal Norm**	ON			
**Social Orientation**	-0.124	0.009	-13.325	< 0.001
**Social Influence**	0.003	0.009	0.273	0.785
**HW Descriptive Norm**	ON			
**Social Orientation**	0.11	0.009	11.857	< 0.001
**Social Influence**	0.024	0.009	2.537	0.011
**SC Descriptive Norm**	ON			
**Social Orientation**	0.247	0.009	27.925	< 0.001
**Social Influence**	-0.015	0.009	-1.658	0.097
**Conscientiousness**	ON			
**Time/Busy**	-0.203	0.01	-20.289	< 0.001
**HW Routine**	0.218	0.01	22.119	< 0.001
**SC Routine**	0.067	0.009	7.45	< 0.001
**Stability**	ON			
**Time/Busy**	-0.098	0.011	-9.23	< 0.001
**HW Routine**	0.131	0.01	12.506	< 0.001
**SC Routine**	0.065	0.009	6.894	< 0.001
**HW Cue**	ON			
**HW Materials**	0.212	0.011	19.65	< 0.001
**SC Materials**	0.248	0.011	23.267	< 0.001
**SC Cue**	ON			
**HW Materials**	0.064	0.011	5.659	< 0.001
**SC Materials**	0.194	0.011	17.671	< 0.001
**HW Automaticity**	ON			
**HW Materials**	0.164	0.011	14.934	< 0.001
**SC Materials**	0.235	0.011	21.81	< 0.001
**SC Automaticity**	ON			
**HW Materials**	-0.089	0.011	-8.082	< 0.001
**SC Materials**	-0.262	0.011	-24.642	< 0.001

**Fig 2:**
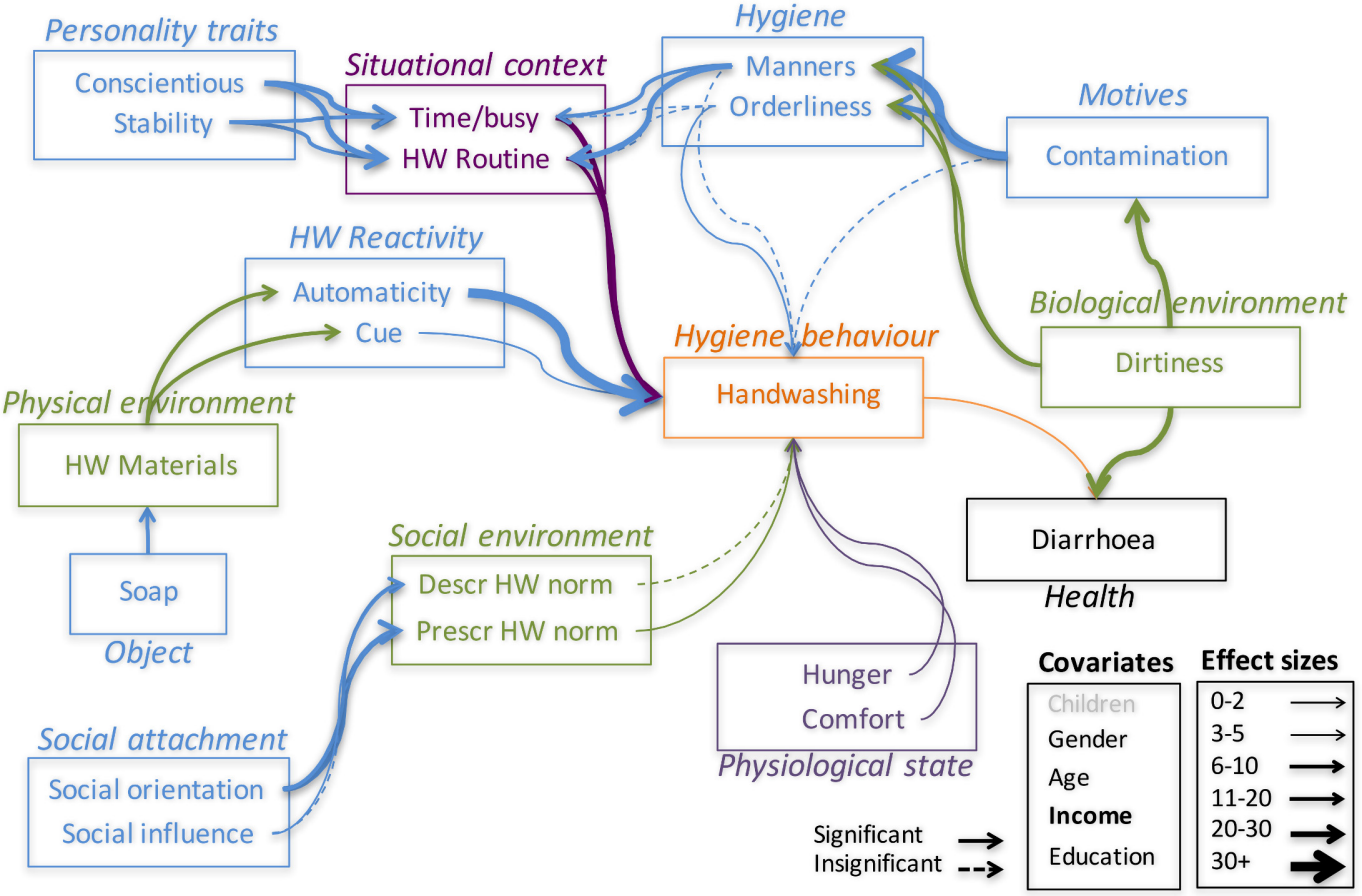
Structural Equation Model Results: Personal Hygiene Factors (only).

**Fig 3:**
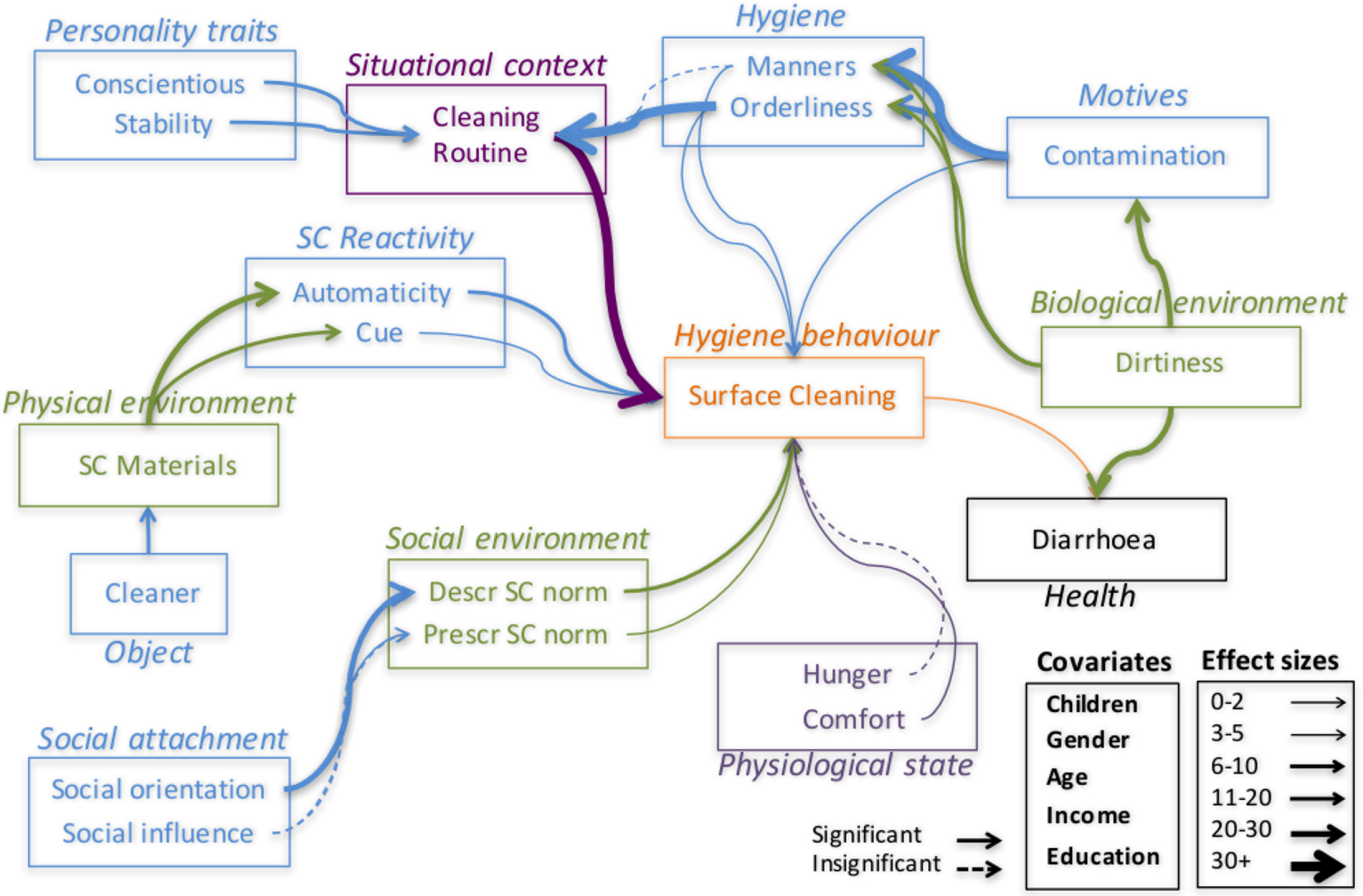
Structural Equation Model Results: Household Hygiene Factors (only).

The structural equation model was estimated with all variables included simultaneously (i.e., for both handwashing and surface cleaning), as this has only small effects on the size of the parameter estimates, and we are interested here only in relative effect sizes. However, in the SEM diagrams below (Figs 2 and 3), results have been depicted independently for handwashing with soap and surface cleaning behaviours, which required duplication of some of the more distal relationships (e.g., between situational and hygiene factors), as these don’t change as a function of which behaviour is being considered. Note that factor values in each row indicate the direction and size of the influence of that factor on the first factor mentioned in each group (in italics). P-values in the table listed as 0 mean p < 0.001. Fig 1 contains a box in which the colour coding scheme is described (e.g., green = environmental factors, black are health factors); this applies to all figures. The variable names themselves use the same colour scheme, so colour only refers to the type of factor.

### Personal Hygiene

Working from the outside (in causal terms) of the SEM results for handwashing with soap (see Fig 2), the first significant result is that positive attitudes toward soap lead individuals to ensure that they have the appropriate materials on hand to enable handwashing–soap and water (*b* = 0.106; p < 0.001). This effect is of middle size (when compared to other effects).

People with the necessary materials in place are more likely to report responding to cues, suggesting that the materials themselves could serve as cues for the behaviour. This is true for both handwashing and surface cleaning (handwashing: *b* = 0.212; p < 0.01; surface cleaning *b* = 0.194; p < 0.01). People with soap and water in their homes also report being more automatic in their handwashing behaviour, as would be expected if responding to soap and water as cues (*b* = 0.164; p < 0.001). This tendency to react to materials as cues, and to practice handwashing automatically are very significantly linked to reported behaviour (*b* = 0.378; p < 0.001).

Turning to the causal stream from Biological Environment, it appears that perceiving oneself as living in an organically Dirty environment has a significant negative impact on infectious disease (*b* = -0.243; p < 0.001). People also respond to such an environment by feeling a greater sense of contamination (*b* = -0.197; p < 0.001).

People responding to the possibility of contamination from a dirty environment see the importance of good manners (i.e., civil acts such as covering a sneeze and not shaking hands with others when their hand is unclean) (*b* = 0.353; p < 0.001). Those sensitive to contamination are also significantly more likely to feel they have to keep their domestic environment tidy (*b* = 0.245; p < 0.001). Good manners in turn makes it less likely that one is dissuaded from handwashing by situational context, in particular being busy or tired (*b* = -0.036; p < 0.001). Mannerly people are also more likely to have a handwashing routine (*b* = 0.224; p < 0.001). However, these hygiene factors, and contamination itself, are not particularly strongly tied to behaviour itself.

On the other hand, people high on the 'Big 5' personality traits of conscientiousness and stability are also more likely to engage in hygienic behaviour, despite being busy (*b* = -0.203; p < 0.001), and to have formed hygiene routines around handwashing (and surface cleaning) (handwashing: (*b* = 0. 218; p < 0.001; surface cleaning: *b* = 0.067; p < 0.001).

Social Orientation is more tightly tied to the various types of norms than is Social Influence by a large margin (Prescriptive Norm: Social Orientation: *b* = -0.256; p < 0.001; Social Influence *b* = -0.016; p = 0.07; Personal Norm: Social Orientation: *b* = -0.124; p < 0.001; Social Influence *b* = 0.003; p = 0.785; Descriptive Norm: Social Orientation: *b* = 0.11; p < 0.001; Social Influence *b* = 0.024; p = 0.011). Prescriptive norms have an impact on behaviour, but descriptive norms do not play a role (Prescriptive Norm: *b* = -0.028; p = 0.002; Descriptive Norm: *b* = -0.008; p = 0.337).

Reported handwashing with soap also has a significant (though small) positive impact on infectious disease health (as measured by frequency of suffering from diarrhoea) (*b* = 0.056; p < 0.001). People who report their environment as being organically dirty also report more frequent diarrhoea (*b* = -0.243; p < 0.001), so there is a significant connection between the possible presence of pathogens and infectious disease in this sample.

There are small, but statistically significant, effects from the Physiological measures on handwashing behaviour too. Being hungry has a small tendency to reduce the likelihood of washing hands (*b* = 0.048; p < 0.001), while people who tend to be bothered by being uncomfortable are more likely to wash their hands (*b* = -0.027; p = 0.001).

Finally, the same demographic effects as seen above in the descriptive statistics study continue to play a role. Women, older, more educated, and particularly more wealthy individuals, are more likely to wash their hands with soap (Gender: *b* = 0.023; p = 0.003; Age: *b* = 0.018; p = 0.03; Education: *b* = 0.022; p = 0.004; Income: *b* = 0.035; p < 0.001).

### Household Hygiene

Our second area of concern is what determines how much respondents engage in household cleaning. In the SEM results, demographic variables are more significant for surface cleaning than handwashing. Fig 3 shows that surface cleaning is conducted more by women than men, especially women with children, and by those with higher incomes and education (Gender: *b* = 0.023; p < 0.001; Age: *b* = 0.084; p < 0.001; Education: *b* = 0.029; p < 0.001; Income: *b* = 0.052; p < 0.001; Children: *b* = -0.059; p < 0.001).

The strongest sequence of links is from dirty environment, to contamination (*b* = -0.197; p < 0.001), to orderliness (*b* = 0.245; p < 0.001), to cleaning routine (*b* = -0.324; p < 0.001), to behaviour (*b* = 0.401; p < 0.001). Beginning with the physical environmental factors, objects have about the same level of significance for both handwashing and surface cleaning (Soap on Handwashing: *b* = 0.106; p < 0.001; Cleaner on Cleaning (*b* = 0.106; p < 0.001).

Looking at the social environment causal stream, there is a chain of significant links from social orientation to descriptive norms to behaviour. The influence of descriptive norms is affected by whether individuals report being supported by others, which influences whether they report others around them being surface cleaners (*b* = -0.059; p < 0.001). If more people in their network are cleaners, then they are more likely to do so too (*b* = 0.055; p < 0.001).

Switching now to the Situational context stream, Personality has small effects on Situational factors (Consciousness: Time/Busy: *b* = -0.203; p < 0.001; SC Routine: *b* = 0.067; p < 0.001; Stability: Time/Busy: *b* = -0.098; p < 0.001; SC Routine *b* = 0.065; p < 0.001). Routine has a very strong effect on whether people report cleaning surfaces (*b* = 0.401; p < 0.001). Manners and contamination also have a significant (if small) independent impact on behaviour (Manners: *b* = -0.019; p < 0.001; Contamination: *b* = 0.059; p < 0.001). If people are uncomfortable, they are more likely to clean surfaces in their house (although the effect is small) (*b* = -0.021; p = 0.011). Finally, surface cleaning has a small, positive impact on reported diarrhoeal disease (*b* = 0.041; p < 0.001).

## Discussion

The SEM results on handwashing with soap suggest that handwashing behaviour is not a particularly strongly motivated behaviour, except in the sense of responding to norms to some degree. It is rather more reactive, although also significantly constrained by situational factors, which mix together psychological and physical barriers to performance.

The results on household cleaning suggest that, among those who clean (who tend to be women with children), the act of cleaning is a regular one, not particularly disturbed by momentary motivations, but not as automatic as handwashing either. Cleaning is part of the daily or weekly routine, in response to a person’s general sense of a need for order in the household environment. Cleaners also pay attention to whether other people in their network are doing the same. It doesn’t matter so much that their friends are pressuring them to clean surfaces; they simply have to notice that it is a common activity in their social circle. Differences in personality also don’t come so much into play–it seems that cleaning routines don’t require the support of conscientiousness or stability to be regularly performed. These routines are not impacted by momentary sensations of hunger or comfort either. This could be because surface cleaning is not so temporally constrained as handwashing–indeed it may happen only once or twice a week, and can be fit into the day at various points, whereas handwashing is more frequent and typically responsive to recent events such as defecation or eating food. Because it is less frequently performed, surface cleaning is less likely to be completely automatic; hence the reduced degree of correlation between reported automaticity and behaviour in this case.

In fact, the biggest difference between surface cleaning and handwashing is that cleaning is about routine, while handwashing is about reactivity and situational effects. The biggest effects on behaviour in the handwashing SEM were automaticity and being busy. In surface cleaning, the top two causes of behaviour are routine and automaticity, with routine being by far the biggest single cause (size of effect: 46.47). Still, there is a good degree of overlap between the SEM results for surface cleaning and handwashing, as might be expected, given that both are cleaning behaviours.

An association between a dirty environment, a sense of contamination and engagement in hygiene behaviours has been widely found in this study. This is consistent with work on disgust and hygiene, which shows that disgust is the natural motive to prompt hygiene behaviour, as it evolved to help people protect themselves from infectious disease threats through avoidance behaviour. [65] Disgust, which underpins a sense of contamination, has been repeatedly found to be an important determinant of hygiene behaviour in empirical studies around the world (although rarely in a multivariate analytical context). [40, 48] What is novel here is the finding that disgust is prompted by the perception of a dirty environment, which sometimes works through other hygiene concerns, like manner and orderliness, to influence behaviour.

Social norms are powerful drivers of most human behaviours–people very commonly behave as they do simply because most others in the relevant social group behave this way. However, norms have not proven to be particularly important in the models examined here. Social norms may not be as important as expected because in countries where hygiene behaviours are endemic, people just grow up knowing they are supposed to do them; good hygiene is below consciousness, and there is little social variation in practice to bring the matter to anyone’s attention. On the other hand, in countries where hygiene is rarely practiced, norms are not strongly associated with the behaviour either–rather, the lack of social pressure to engage in these behaviours makes the connection between norms and behaviour weak.

A couple of ‘Big 5’ personality traits, stability and particularly conscientiousness, turned out to be important dimensions of variation in the causal structure of hygiene behaviour. Conscientiousness is associated with people who are careful, painstaking, deliberate, thorough, organized, goal-oriented, and able to control their own impulses. (It is already well-known that conscientious people are organized, and hence high on orderliness–indeed, orderliness is often considered a ‘facet’ or dimension of conscientiousness [66, 67]–although it is not included in the measurement of the trait in the dominant Big Five scale. People high in conscientiousness like to live according to routines and schedules; they are known to keep lists and to make plans prior to action.) This result reinforces the significance found for habit and routine in determining these hygiene behaviours. It is interesting that Personality seems to work on behaviour via Situational context variables, suggesting that they play a role in helping people to form habits and routines by ensuring regular practice.

It is clear that people who find hygiene products more intrinsically appealing are more likely to have these products in their domestic environs, which in turn facilitates the related behaviours being performed more regularly. We also found (in questionnaire responses not included in the SEM analysis), that the reasons people have hygiene products in their homes in the first place can be varied: they can be considered purifying, as something in wide use, protective against germs, a way of caring for others, or comforting. But it is also difficult for people to think of these products in certain kinds of ways–e.g., as a badge of membership in a group, status symbol, something to play with, or a sexy or disgusting thing. These limitations suggest that people more naturally associate hygiene products with their functional use, or with something that people in their social networks have (and presumably use), or as a way of helping protect their family members. As everyday products, they don’t have much caché in terms of social status, and are difficult to think of except in functional terms.

Situational factors also play a major explanatory role in both of the hygiene behaviours we examined. People are perturbed by being busy, or tired, and hence not wanting to engage in hygienic behaviour (particularly handwashing). This kind of ‘in the moment’ constraint is seldom considered in behavioural models, but has proven to be significant for these everyday behaviours. This result suggests that people sometimes find it difficult to manage their overall plan of activities for the day to ensure that they are able to conduct this important behaviour when it becomes necessary. In particular, the huge significance of routine in the surface cleaning model implies that people must find ways to ensure that they build up cleaning routines for this behaviour in order for it to be regularly performed. Results with respect to dental care behaviour suggests that making a new behaviour part of the daily routine depends on where in the stream of activities this behaviour is inserted: in particular, flossing behaviour became more reliable if performed after, rather than before, tooth-brushing. [49] A previous study also showed that having the facilities handy was associated with having clean hands in a rural Bangladeshi population. [68] Similar constraints might play a role in forming regular routines for cleaning surfaces around the house.

There are a number of reasons to be circumspect about the significance of the results. First, the measures of model fit are poor. We note that for CFI and TLI values >0.90 are indicative of acceptable fit, whereas for the RMSEA values <0.06 indicate good fit. However, actual values are considerably lower than these. We believe this is because this is a test of a particular theory’s ability to account for global data, not a purely empirical attempt to recover the most meaningful set of parameters. As a consequence, model fit statistics are not as high as a purely empirical estimation attempt might have uncovered. We can conclude that the Behaviour Determination Model is not a particularly powerful predictor of variation in reported hygiene practices.

Second, questionaire-based studies have obvious limitations in that outcome variables–in this case both behavioural and disease-related–are only reported, rather than observed. This can lead to significant biases in responding, typically to favour more normative response categories. Biases. [60, 69–71] Nevertheless, this method has been pursued for this study because the objective is not to accurately measure levels of outcomes, but rather to apportion variability in outcomes to causal factors such as environmental conditions and psychological traits. If one assumes that reporting biases are consistent across populations, then reported data should be sufficient to fulfill this objective.

Third, many of the variables which have proven most significant are explicitly about hygiene. (In measurement terms, they make explicit reference to hygiene-related phenomena, such as continuing to handwash even when busy.) These variables include automaticity, routine, hunger, materials, contamination, and the norm measures. This suggests that these variables have had an ‘unfair advantage’ in the model estimation process: they are more likely to be significantly correlated with hygiene behaviour simply because they cover the same kind of phenomenon: hygiene. The other variables have the disadvantage of being more general, being about personality, or the nature of the social or biological world (e.g., living in a dirty environment). This suggests that a truer picture of the relative importance of relationships would be had by downgrading or handicapping the explicitly hygienic variables to enable a fairer playing field. Finding a means of doing this is exceedingly difficult, however.

Fourth, given the large sample size, many relationships are likely to be statistically significant. For this reason, we have concentrated here on effect sizes, not statistical probabilities, since the more interesting question in this context is which relationships are most important in causing the outcomes of behaviour or health. It is also the case that in a structured equation model which defines only a limited number from among all possible relationships among variables, and those based on theoretical reasoning, that spuriously significant relationships are less likely to arise.

Fifth, the Behaviour Determination Model allows the examination of a broad range of factors on frequent performance of an everyday behaviour, or the mediators of such performance, including psychological traits, environmental triggers and social factors. Even so, the ability to find strong determinants of behaviour has been limited. There are several possible interpretations of the relatively weak correlations with hygiene behaviour in the results:

- Reported behaviour (as here) is a poor measure of actual behaviour
- The important causes of behaviour have been left out of the model
- Behaviour has multiple determinants, each with relatively small effect (i.e., the model is a good representation of the facts)

Unfortunately, we are not in position to discriminate among these alternative explanations. We certainly know from other work that the first explanation is likely; many studies have shown the discrepancies between reported and actual behaviour. [59–61] However, this study is not about measuring actual levels of behaviour or disease, but rather the relative importance of causal pathways associated with behaviour and disease, which can be based on variation in the kinds of data reported. By concentrating only on differences in the relative importance of factors correlated with reported behaviour and disease, assuming that large numbers of respondents wash away bias, we can reliably report on effects. The second explanation seems implausible, given that we have included such a wide range of variables in the model; however, each of them could be poor measures of those kinds of causes, so we can’t really exclude that explanation either. It is probable that the third explanation is true as well, but again, we have no independent grounds beyond plausibility for saying so.

Nevertheless, there are reasons to believe in the general validity of the results. First, the SEM can be considered a model of the *correlates* of current behaviour (as component questions in the measure of behaviour are concerned with how often the behaviour is performed, taken as a more reliable estimate of whether the report of engaging in the behaviour is actually true). This perspective on the model makes it even more clear why measures of automaticity and routine should figure so prominently among the determinants of this measure of behaviour, as habitualness and frequency of performance are tightly linked: automaticity can only be reported (honestly) for an often-enacted behaviour. In fact, it is almost impossible for someone to report performing handwashing behaviour automatically without having already done so a large number of times, which suggests that the correlation between such a report and reported behaviour is bound to be large. But it also likely reflects the reality: such everyday behaviours tend to become automatic.

## Conclusion

Here, we have estimated a structural equation model of personal and household hygiene behaviour from a large, globally representative sample. The most significant conclusion from this modeling exercise is that these everyday behaviours are largely performed automatically in response to environmental cues (including a Dirty environment). However, the SEM also indicates that this practice can be interrupted by being too busy, or hungry. So situational factors still seem to intervene to some degree, even when behaviours have become automatic (although surface cleaning is less situationally constrained than handwashing, both logically, and in the SEM results). The primary implication of this study in practical terms is thus that psychologists and public health workers should look more closely at non-cognitive causes of behaviour, especially if those behaviours are performed regularly.

We have also shown the importance of a number of factors not previously considered in studies of hygiene behaviours at this scale–in particular, the roles of manners, orderliness and routine, suggesting their general significance. Taken together with the results on habitual performance, we hope that these insights will be taken into consideration when developing behaviour change campaigns promoting these health-related behaviours around the world.

## Appendix 1: The Survey Questionnaire

### Demographic questions

DQ1. Please specify your gender

Please tick one answer only.

■ Male
■ Female

DQ2.Please specify your age

Please tick one answer only.

■ Drop box with ages

DQ3.What is your relationship status?

■ Single parent with dependent children (hide fifth option of DQ4)
■ Single parent with non dependent children (hide fifth option of DQ4)
■ With partner / married and dependent children (hide fifth option of DQ4)
■ With partner / married and non dependent children (hide fifth option of DQ4)
■ Single, no children (Skip DQ4 BUT add respondents into ‘I do not have any children’ at reporting
■ With partner / married no children (Skip DQ4 BUT add respondents into ‘I do not have any children’ at reporting
■ Other

DQ4. How old are your children?

(Tick all that apply)

■ 0–5
■ 6–11
■ 12–17
■ 18+
■ I do not have children

DQ5. How many people are in your household?

■ 1
■ 2
■ 3
■ 4
■ 5
■ 6
■ 7
■ 8
■ 9
■ 10+

DQ6. Are you currently in paid employment?

Please tick one answer only.

■ Yes, full time (hide sixth option of DQ8)
■ Yes, part time (hide sixth option of DQ8)
■ No (hide first, second, third and eight options of DQ8)

**DQ7. What is your average annual household income?** (Adapt for each country)

Please tick one answer only.

DQ8. Please tick the option that best describes your current profession.

Please tick one answer only.

■ Manual worker (blue-collar)
■ Employee in an office (white-collar)
■ Civil servant
■ Retired
■ Homemaker
■ Presently unemployed
■ Student/pupil
■ Informal work (self-employed, ad hoc employment or small scale entrepreneur)
■ Other

**DQ9. What is your highest level of formal education?** (Adapt for each country)

Please tick one answer only.

■ No formal qualifications
■ Attended primary school
■ Attended secondary school (GCSE / O levels)
■ Attended 6th form (A levels)
■ Attended college/ vocational education (non-degree level)
■ Attended university (degree)
■ Attended post-graduate qualifications
■ Other

**DQ10. Which region do you live in?** (Adapt for each country)

Please tick one answer only.

DQ11. Our household owns (tick all that apply):

■ The structure in which we live
■ Land for farming
■ Animals that we intend to eat or use for work (i.e., not pets)
■ A car
■ A water tap that works inside the house
■ A refrigerator
■ A freezer
■ A toilet
■ None of the above

### Survey questions

1. Yesterday, food was prepared in my household (check all that apply):

■ Outside the house
■ On the floor inside the house
■ On surfaces inside the house such as the kitchen table, the kitchen counter top, a wooden or plastic board
■ I didn’t prepare food in my house yesterday

2. The neighbourhood where I live is quite dirty.

(Strongly agree, agree, neutral, disagree, strongly disagree)

3. If you walk around the neighbourhood where I live, you will see human or animal faeces on the ground.

(Strongly agree, agree, neutral, disagree, strongly disagree)

4. I don’t have a strong group of friends who help me if I have a problem.

(Strongly agree, agree, neutral, disagree, strongly disagree)

5. I don’t often interact with other people.

(Strongly agree, agree, neutral, disagree, strongly disagree)

6. I do what I like; I really do not care what other people say about me.

(Strongly agree, agree, neutral, disagree, strongly disagree)

7. In general, I want to do what the people who are important to me think I should do.

(Strongly agree, agree, neutral, disagree, strongly disagree)

8. My friends and I often talk about how clean people are.

(Strongly agree, agree, neutral, disagree, strongly disagree)

9. My friends and I often talk about how tidy people keep their houses.

(Strongly agree, agree, neutral, disagree, strongly disagree)

10. **My friends and I often talk about how clean people keep the kitchen and bathroom surfaces in their houses.** (Strongly agree, agree, neutral, disagree, strongly disagree)

11. I would say that cleaning household surfaces is something we practice a lot round here.

(Strongly agree, agree, neutral, disagree, strongly disagree)

12. **I would say that hand-washing with soap is** **not** **something we practice much round here.** (Strongly agree, agree, neutral, disagree, strongly disagree)

13. I would say that hand-washing with an antibacterial product is not something we practice much around here.

(Strongly agree, agree, neutral, disagree, strongly disagree)

14. Most of the people important to me don’t care if I wash my hands with soap.

(Strongly agree, agree, neutral, disagree, strongly disagree)

15. Most of the people important to me think I should keep my household surfaces clean.

(Strongly agree, agree, neutral, disagree, strongly disagree)

16. I would feel guilty if I didn’t wash my hands with soap after using the toilet.

(Strongly agree, agree, neutral, disagree, strongly disagree)

17. I feel a strong personal obligation to wash my hands with soap.

(Strongly agree, agree, neutral, disagree, strongly disagree)

18. Even when I am tired, I manage to wash my hands with soap after the toilet.

(Strongly agree, agree, neutral, disagree, strongly disagree)

19. I would wash my hands with soap more often if I wasn’t often doing other things at the same time.

(Strongly agree, agree, neutral, disagree, strongly disagree)

20. Even if I am busy, I manage to wash my hands with soap after the toilet.

(Strongly agree, agree, neutral, disagree, strongly disagree)

21. It takes too much time to wash my hands with soap each time I prepare food.

(Strongly agree, agree, neutral, disagree, strongly disagree)

22. It takes too much time to wash kitchen surfaces with a cleaning product each time I use them to prepare food.

(Strongly agree, agree, neutral, disagree, strongly disagree)

23. Hand-washing with soap is quick and easy to do.

(Strongly agree, agree, neutral, disagree, strongly disagree)

24. All the things I need to wash my hands with soap are readily available in my house (i.e. clean water and soap).

(Strongly agree, agree, neutral, disagree, strongly disagree)

25. All the things I need to clean surfaces are readily available in my house (i.e. cleaning liquid and a cloth).

(Strongly agree, agree, neutral, disagree, strongly disagree)

26. I find soap affordable.

(Strongly agree, agree, neutral, disagree, strongly disagree)

27. I find antibacterial hand wash affordable.

(Strongly agree, agree, neutral, disagree, strongly disagree)

28. I find household cleaning products affordable.

(Strongly agree, agree, neutral, disagree, strongly disagree)

29. If I am hungry, I often don’t bother to wash my hands before eating.

(Strongly agree, agree, neutral, disagree, strongly disagree)

30. I like to go through the same sequence of activities every day.

(Strongly agree, agree, neutral, disagree, strongly disagree)

31. For me, what I do one day is very much like the next.

(Strongly agree, agree, neutral, disagree, strongly disagree)

32. I can easily go through my daily routine without washing my hands with soap.

(Strongly agree, agree, neutral, disagree, strongly disagree)

33. It’s easy to forget to wash your hands before eating food.

(Strongly agree, agree, neutral, disagree, strongly disagree)

34. I try to make sure I do a bit of cleaning around the house every day.

(Strongly agree, agree, neutral, disagree, strongly disagree)

35. I try to clean the kitchen thoroughly (please check one option that reflects your practice)

■ Every day
■ Once a week
■ Once every two weeks
■ Once a month
■ Less often than once a month

36. I try to clean the bathroom thoroughly (please check one option that reflects your practice)

■ Every day
■ Once a week
■ Once every two weeks
■ Once a month
■ Less often than once a month

You should rate the extent to which the pair of traits applies to you, even if one characteristic applies more strongly than the other.

I see myself as:

37. Extraverted, enthusiastic.

(Strongly agree, agree, neutral, disagree, strongly disagree)

38. Critical, quarrelsome.

(Strongly agree, agree, neutral, disagree, strongly disagree)

39. Dependable, self-disciplined.

(Strongly agree, agree, neutral, disagree, strongly disagree)

40. Anxious, easily upset.

(Strongly agree, agree, neutral, disagree, strongly disagree)

41. Open to new experiences, complex.

(Strongly agree, agree, neutral, disagree, strongly disagree)

42. Reserved, quiet.

(Strongly agree, agree, neutral, disagree, strongly disagree)

43. Sympathetic, warm.

(Strongly agree, agree, neutral, disagree, strongly disagree)

44. Disorganized, careless.

(Strongly agree, agree, neutral, disagree, strongly disagree)

45. Calm, emotionally stable.

(Strongly agree, agree, neutral, disagree, strongly disagree)

46. Conventional, uncreative.

(Strongly agree, agree, neutral, disagree, strongly disagree)

47. I rarely organize my day to make sure that all the most important things get done.

(Strongly agree, agree, neutral, disagree, strongly disagree)

48. I find it hard to get started on big projects that require several different steps.

(Strongly agree, agree, neutral, disagree, strongly disagree)

49. I am not easily distracted from completing unpleasant tasks.

(Strongly agree, agree, neutral, disagree, strongly disagree)

50. If I am interrupted, it is easy for me to get started again where I left off.

(Strongly agree, agree, neutral, disagree, strongly disagree)

51. I avoid people who look ill.

(Strongly agree, agree, neutral, disagree, strongly disagree)

52. I don’t think of myself as a squeamish person.

(Strongly agree, agree, neutral, disagree, strongly disagree)

**53. I would never stop being friends with someone because of their immoral behaviour.** (Strongly agree, agree, neutral, disagree, strongly disagree)

54. When I think something is unfair I have to do something about it.

(Strongly agree, agree, neutral, disagree, strongly disagree)

**55. I would usually rather do something at home on my own than go out to a social event.** (Strongly agree, agree, neutral, disagree, strongly disagree)

56. When meeting a new group of people I’m anxious to figure out how to behave in order to fit in as quickly as possible.

(Strongly agree, agree, neutral, disagree, strongly disagree)

57. I don’t crave recognition whenever I achieve a goal.

(Strongly agree, agree, neutral, disagree, strongly disagree)

58. Maintaining a good reputation is the most important thing in life.

(Strongly agree, agree, neutral, disagree, strongly disagree)

59. I’m not bothered by feeling sweaty and sticky.

(Strongly agree, agree, neutral, disagree, strongly disagree)

60. I don’t cope well with pain.

(Strongly agree, agree, neutral, disagree, strongly disagree)

61. Being a parent is the most important role one can play in life.

(Strongly agree, agree, neutral, disagree, strongly disagree)

62. I am happiest when caring for others.

(Strongly agree, agree, neutral, disagree, strongly disagree)

63. I don’t like the feeling of my hands after they have been washed with soap.

(Strongly agree, agree, neutral, disagree, strongly disagree)

64. I think my house becomes more beautiful when I clean kitchen and bathroom surfaces.

(Strongly agree, agree, neutral, disagree, strongly disagree)

**65. I sometimes start washing my hands with soap without even realizing I'm doing it.** (Strongly agree, agree, neutral, disagree, strongly disagree)

66. I feel strange when I don’t wash my hands with soap after the toilet.

(Strongly agree, agree, neutral, disagree, strongly disagree)

**67. Washing my hands with soap before I eat a meal is something I do automatically.** (Strongly agree, agree, neutral, disagree, strongly disagree)

68. I began washing my hands with soap just recently.

(Strongly agree, agree, neutral, disagree, strongly disagree)

69. Seeing soap after having been to toilet makes me wash my hands.

(Strongly agree, agree, neutral, disagree, strongly disagree)

70. A bad smell or visible dirt on my hands makes me want to wash them with soap.

(Strongly agree, agree, neutral, disagree, strongly disagree)

71. I sometimes start cleaning my house without even realizing I'm doing it.

(Strongly agree, agree, neutral, disagree, strongly disagree)

72. I feel strange when I don’t clean my house regularly.

(Strongly agree, agree, neutral, disagree, strongly disagree)

73. Washing the kitchen surface or chopping board with a cleaning product before I prepare food or eat a meal is something I do automatically.

(Strongly agree, agree, neutral, disagree, strongly disagree)

74. I started regularly cleaning the place I live in a long time ago.

(Strongly agree, agree, neutral, disagree, strongly disagree)

**75. Seeing a bit of dirt on a household surface makes me want to clean it immediately.** (Strongly agree, agree, neutral, disagree, strongly disagree)

76. I have heard of antibacterial cleaning products

■ Yes
■ No [skip Q77]

77. Please select one answer

■ I sometimes prefer to use an antibacterial cleaning product over regular cleaning products when cleaning my house.
■ I always prefer to use an antibacterial cleaning product over regular cleaning products when cleaning my house.
■ I don’t think about the benefits an antibacterial cleaner might have over a regular cleaner when cleaning my house.
■ I think about the benefits of an antibacterial cleaner but I choose not to use one

78. I have heard of antibacterial soap.

■ Yes
■ No [skip Q79 / Q80]

79. I have antibacterial soap in my house right now.

(Strongly agree, agree, neutral, disagree, strongly disagree)

80. Please select one answer

■ I sometimes prefer to use an antibacterial soap over regular soap when washing my hands.
■ I always prefer to use an antibacterial soap over regular soap when washing my hands.
■ I don’t think about the benefits an antibacterial soap might have over regular soap when washing my hands.
■ I think about the benefits of an antibacterial soap but I choose not to use one

81. I often decide to do something in a few minutes time, but then forget to do it.

(Strongly agree, agree, neutral, disagree, strongly disagree)

82. Soap can be:

(Tick all that apply)

■ Disgusting
■ Comforting
■ Sexy
■ Purifying
■ A store of economic value
■ An indicator of social status
■ A means of caring for others
■ Something everyone uses
■ An object to play with
■ Something that helps me protect others from my germs
■ A badge of belonging to a social group
■ Other

83. Surface cleaning liquid can be:

(Tick all that apply)

■ Disgusting
■ Comforting
■ Sexy
■ Purifying
■ A store of economic value
■ An indicator of social status
■ A means of caring for others
■ Something everyone uses
■ An object to play with
■ Something that helps me protect others from my germs
■ A badge of belonging to a social group
■ Other

84. If a home does not look dirty then it doesn’t need cleaning.

(Strongly agree, agree, neutral, disagree, strongly disagree)

85. Hidden germs cause diarrhea.

(Strongly agree, agree, neutral, disagree, strongly disagree)

86. After using the toilet there may be unseen contamination on my hands.

(Strongly agree, agree, neutral, disagree, strongly disagree)

87. You only need to wash your hands when they look or feel dirty.

(Strongly agree, agree, neutral, disagree, strongly disagree)

88. Using an antibacterial product to wash your hands does not provide a benefit over soap in regard to cleanliness

(Strongly agree, agree, neutral, disagree, strongly disagree)

**89. Hand washing technique is important to ensure all contamination is removed from hands.** (Strongly agree, agree, neutral, disagree, strongly disagree)

90. I don’t feel uncomfortable if my surroundings are messy.

(Strongly agree, agree, neutral, disagree, strongly disagree)

91. If I see things in a jumble or out of place, I feel compelled to put them in order.

(Strongly agree, agree, neutral, disagree, strongly disagree)

**92. I have a habit of constantly organizing the things in my house or at my work-place.** (Strongly agree, agree, neutral, disagree, strongly disagree)

93. I am a very tidy person.

(Strongly agree, agree, neutral, disagree, strongly disagree)

94. I would shake hands knowing that my hands are dirty.

(Strongly agree, agree, neutral, disagree, strongly disagree)

95. As a guest in someone else’s house, I always make sure I clean up the room after using their toilet.

(Strongly agree, agree, neutral, disagree, strongly disagree)

96. I would not feel embarrassed if I sneezed or coughed in front of someone without covering my mouth.

(Strongly agree, agree, neutral, disagree, strongly disagree)

97. I washed my hands with soap ___ times yesterday.

■ 0
■ 1–2
■ 3–4
■ 5–6
■ > 6

98. I wash my hands with soap after using the toilet.

■ Always
■ Often
■ Sometimes,
■ Rarely
■ Never

99. I wash my hands with soap before preparing or eating food.

■ Always
■ Often
■ Sometimes,
■ Rarely
■ Never

100. I often use a hand sanitizer when soap and water are not available.

(Strongly agree, agree, neutral, disagree, strongly disagree)

101. I spent time removing dirt from different places in my house yesterday.

(Strongly agree, agree, neutral, disagree, strongly disagree)

102. I wipe kitchen and bathroom surfaces clean around my house

■ Every day
■ Every other day
■ Once a week
■ Rarely
■ Never

103. I use a cleaning product when I tidy my house.

(Strongly agree, agree, neutral, disagree, strongly disagree)

104. I used a surface cleaner ____ times in the past week.

■ 0
■ 1–2
■ 3–4
■ 5–6
■ > 6

105. I have many different cleaning products in my house.

■ Yes
■ No

106. I often have colds.

(Strongly agree, agree, neutral, disagree, strongly disagree)

107. I never have skin infections.

(Strongly agree, agree, neutral, disagree, strongly disagree)

108. I often get diarrhea.

(Strongly agree, agree, neutral, disagree, strongly disagree)

109. I was often ill when I was young.

(Strongly agree, agree, neutral, disagree, strongly disagree)

110. I feel I am more susceptible than most people to falling ill to infections.

(Strongly agree, agree, neutral, disagree, strongly disagree)

111. I have a strong constitution and so rarely get ill.

(Strongly agree, agree, neutral, disagree, strongly disagree)

112. My immune system protects me from most illnesses that other people get.

(Strongly agree, agree, neutral, disagree, strongly disagree)

113. I manage to stay healthy when others around me are ill.

(Strongly agree, agree, neutral, disagree, strongly disagree)

114. I have had to leave off doing my normal course of work due to infectious illness for ___ days in the past year.

■ 0
■ 1–3
■ 4–7
■ 8–14
■ > 14

115.

(Yes/No)

■ I have diabetes.
■ I have suffered from malaria.
■ I have never had tuberculosis.

116. If a child gets diarrhoea, it will not have a severe impact on their health.

(Strongly agree, agree, neutral, disagree, strongly disagree)

117. If I get diarrhoea, it could have a severe impact on my health.

(Strongly agree, agree, neutral, disagree, strongly disagree)

118. I intend to wash my hands with soap every time I go to the toilet.

(Strongly agree, agree, neutral, disagree, strongly disagree)

119. If I wanted to, I could wash my hands with soap every time I defecate.

(Strongly agree, agree, neutral, disagree, strongly disagree)
